# Supplementary material for: Preferential selection of viral escape mutants by CD8+ T cell ‘sieving’ of SIV reactivation from latency
Source: PLoS Pathog. 2023 Nov 30;19(11):e1011755. doi: 10.1371/journal.ppat.1011755 (PMC10688670; doi:10.1371/journal.ppat.1011755)
Supplement: S1 Text — (DOCX) [file ppat.1011755.s001.docx]

1. Limit of detection for Illumina sequencing

Sequencing errors and in vitro recombination are relatively common in Illumina sequencing. Therefore, limits of detection were set for quantification of barcodes, Tat-SL8 variants, and barcode-Tat-SL8 linkages.

In this analysis, output reads were discarded if the barcode did not match a barcode from the previous stock characterization, the Tat-SL8 epitope was not detected, or an indel was detected in the Tat-SL8 epitope. Such defective reads were also not included when quantifying the total number of output reads.

- 1. Barcode limit of detection

To ensure barcodes observed in the sequencing analysis were genuine and present on at least one template in the sample, the limit of detection for barcodes was set based on the amplification factor of the sequencing run. The amplification factor, $A$, was defined as the ratio of number of reads to number of templates. For each sequencing run, the limit of detection was set to $\max\left( A,2 \right)$. The limit of detection was 2 in sequencing runs with $A<2$ so as to ensure a single sequencing error would not cause a false barcode read to be registered.

- 1. Tat-SL8 variant limit of detection

Similar to as with barcodes, Tat-SL8 variants with read counts of less than 2 or the amplification factor were deemed below the detection threshold. Additionally, we accounted for Tat-SL8 epitopes being generated at a low rate in vitro during bulk PCR by implementing a proportional cut-off at 10^-2.54^ of the total number of reads. This proportional cut-off is the most conservative of the cut-offs used by Immonen et al. 2020 (1), which are based on their direct measurement of the generation of 13 Tat-SL8 variants during bulk sequencing (1). In summary, for each sequencing run, the limit of detection for Tat-SL8 variants was $\max\left( A,2,S\times{10}^{-2.54} \right)$, where $S$ is the total number of reads.

- 1. Barcode-Tat-SL8 combination limit of detection

Finally, similar to total Tat-SL8 variant quantification, Tat-SL8 variants on individual barcodes were required to consist of at least 2 reads, the amplification factor, and ${10}^{-2.54}$ fold of the total number of reads of the *barcode* to be above the detection threshold. In vitro recombination was also taken into account as Immonen et al. 2020 demonstrated that in vitro recombination occurs at a rate of 13% during bulk PCR (1). Thus, for a barcode-Tat-SL8 variant linkage to be deemed genuine, the fraction of the barcode reads containing the Tat-SL8 variant was required to be greater than 13% of the fraction the Tat-SL8 variant constituted in the overall viral load. In short, for each sequencing run, the limit of detection for the linkage of barcode $i$ and Tat-SL8 variant $k$ was $\max\left( A,2,B_{i}\times{10}^{-2.54}, .13\times v_{k}\times B_{i} \right)$, where $B_{i}$ is the total number of reads of barcode $i$ and $v_{k}$ is the fraction of total reads that were Tat-SL8 variant $k$.

References

1. Immonen TT, Camus C, Reid C, Fennessey CM, Del Prete GQ, Davenport MP, et al. Genetically barcoded SIV reveals the emergence of escape mutations in multiple viral lineages during immune escape. Proc Natl Acad Sci U S A. 2020;117(1):494-502.
