# Supplementary material for: Preferential selection of viral escape mutants by CD8+ T cell ‘sieving’ of SIV reactivation from latency
Source: PLoS Pathog. 2023 Nov 30;19(11):e1011755. doi: 10.1371/journal.ppat.1011755 (PMC10688670; doi:10.1371/journal.ppat.1011755)
Supplement: S2 Text — (DOCX) [file ppat.1011755.s002.docx]

1. Tat-SL8-specific CD8^+^ T cell phenotype flow cytometry analysis

The following antibodies were obtained from BD Biosciences: Granzyme B AF700 (clone GB11), CXCR3 BUV395 (clone IC6/CXCR3) and KI67 BV785 (clone B56); from MabTech: Perforin FITC (clone pf344); from eBioscience: CXCR5 PE-Cy7 (clone MU5UBEE) and T-bet PerCP-Cy5.5 (clone 4B10); from Biolegend: CD69 BV605 (clone FN50), PD1 BV711 (clone EH12.2H7) and CX3CR1 PE (clone KO124E1).
