# Supplementary material for: Preferential selection of viral escape mutants by CD8+ T cell ‘sieving’ of SIV reactivation from latency
Source: PLoS Pathog. 2023 Nov 30;19(11):e1011755. doi: 10.1371/journal.ppat.1011755 (PMC10688670; doi:10.1371/journal.ppat.1011755)
Supplement: S3 Text — (DOCX) [file ppat.1011755.s003.docx]

1. Modeling predictors of variant reactivation at second treatment interruption
   1. Introduction

In the main text, we demonstrate that early after the second treatment interruption, the majority of plasma viral load is escaped at the Tat-SL8 epitope. We also provide anecdotal evidence that the escape variants dominating viral load early post rebound reactivated from the reservoir. In this Supplement, we seek to confirm that after the second rebound, early dominance by escaped virus is driven by reactivation of escaped variants from the reservoir as opposed to some other mechanism. To this end, we consider all possible mechanisms by which individual escape variants may arise among replicating virus and determine which hypothesis best matches the observed data on variants detected early after rebound.

The potential mechanisms that may have determined which escape variants were present early after the second treatment interruption are; 1) the collection of variants in the viral load was completely random, 2) individual variants were present in the reservoir, and larger variants in the reservoir were more likely to reactivate, 3) variants evolved from wild-type virus after reactivation, and variants consisting of more probable mutations were more likely to evolve, and therefore be detected in the viral plasma, or 4) a combination of the above 3 mechanisms are at play.

- 1. Model

To test which if any of these hypotheses are best supported by the data, we first develop mathematical models based on each hypothesis to predict the collection of variants in the viral load after rebound. We then explain how the data is incorporated into the models. Finally, we present the results of fitting the various models to data from each animal and show the calculated corrected Akaike Information Criterion (AIC) (1) and results of the likelihood ratio tests to examine which model best predicts variants detected early after treatment interruption.

We begin by defining the probability density function for the appearance of a variant in an animal after a period of time. To do this we first define $t$ as the time since ART release, and $w$ to be the drug washout period following removal of ART. Therefore, the duration of time during which an individual Tat-SL8 variant could reactivate or evolve is given by $t-w$. Additionally, we assume the time at which a variant appears among the plasma virus is exponentially distributed. We note that the rate at which variant $k$ appears among the plasma virus of animal $j$ after ART release will depend on a number of variant and animal specific variables and parameters, and so in actuality will be a function of these parameters and variables. However, for simplicity of notation we will represent it as a variable with subscripts $j$ and $k$ and only later define its functional form. Therefore, we define $\tilde{r}_{k,j}$ to be the rate at which variant $k$ appears among the plasma virus of animal $j$ after ART release.

The exponential probability density function (p.d.f.) for the appearance of variant $k$ in animal $j$ at time $t$, $p_{k,j}(t)$, is given by

$$\begin{aligned} {p_{k,j}\left( t \right)=\tilde{r}}_{k,j}e^{-\tilde{r}_{k,j}\left( t-w \right)}\# \end{aligned}(S3.1)$$

and the cumulative distribution function (c.d.f.) for appearance by time $t$, $c_{k,j}(t)$, is

$$\begin{aligned} c_{k,j}\left( t \right)=\int_{w}^{t} p_{k,j}\left( s \right)ds=\int_{w}^{t} \tilde{r}_{k,j}e^{-\tilde{r}_{k,j}\left( s-w \right)}ds=-e^{-\tilde{r}_{k,j}\left( s-w \right)}│\begin{matrix} t \\ \\ w \end{matrix}=1-e^{-\tilde{r}_{k,j}\left( t-w \right)}.\# \end{aligned}(S3.2)$$

We next define $t_{j}$ as the time at which sequencing occurred in animal $j$. Then ${z_{k,j}=c}_{k,j}\left( t_{j} \right)$ gives the probability of detecting variant $k$ in animal $j$ at the time of sequencing and is given by:

$$\begin{aligned} z_{k,j}=c_{k,j}\left( t_{j} \right)=1-e^{-\tilde{r}_{k,j}\left( t_{j}-w \right)}=1-e^{-r_{k,j}}\# \end{aligned}(S3.3)$$

where $r_{k,j}=\tilde{r}_{k,j}\left( t_{j}-w \right)$.

To test which of the potential mechanisms for the emergence of individual variants after rebound outlined in the Introduction of this Supplement (Section S3.1) best matches the data, we formulate mathematical models that correspond to each of the four hypotheses. Within these hypotheses, we allow $r_{k,j}$ to be a function of both variant specific and animal specific variables, $\vec{x}_{k,j}$, and animal specific parameters, $\vec{\theta}_{j}$, and allow $r_{k,j}\left( \vec{x}_{k,j};\vec{\theta}_{j} \right)$ to take on a different form depending on the hypothesis being considered.

- Constant Model: The simplest model corresponds to the first hypothesis listed in S3.1; all variants within an animal have equal probability of being detected at the time of sequencing. This is achieved when $r_{k,j}$ is constant across all variants in animal $j$, i.e.

$\begin{aligned} r_{k,j}=r_{j}\# \end{aligned}(S3.4)$

- Reservoir Model: This model assumes that the probability of detecting a variant is dependent on the amount of the variant present in the reservoir. We assume there is a baseline contribution to this probability for each animal ($r_{j}$), and that for individual variants, the probability increases above this baseline level based on the amount of variant $k$ in the reservoir of animal $j$ (denoted by $q_{k,j}$), and an animal dependent constant,$b_{j}$. Thus, for this model $r_{k,j}$ is defined as in Equation $\left( S3.5 \right)$below.

$\begin{aligned} r_{k,j}=r_{j}+b_{j}q_{k,j}\# \end{aligned}(S3.5)$

- Evolution Model: This model assumes that the probability of detecting a variant is based on the chance it evolves from the wild-type variant after reactivation. Once again, there is a baseline contribution to the probability of detection for each animal ($r_{j}$) and the probability of detection is also determined based on an animal specific mutation rate factor ($m_{j}$) and a variant specific propensity for evolution, $f_{k}$. This leads to $r_{k,j}$ being defined as:

$\begin{aligned} r_{k,j}=r_{j}+m_{j}f_{k}\# \end{aligned}(S3.6)$

- Combined Model: Finally, we consider a model where both the reservoir composition and propensity for evolution combine to impact the probability that a variant is detected. This model is a composite of the Reservoir Model and the Evolution Model. In this model $r_{k,j}$ is given by

$\begin{aligned} r_{k,j}=r_{j}+b_{j}q_{k,j}+m_{j}f_{k}\# \end{aligned}(S3.7)$

Now that we have outlined the functional forms for $r_{k,j}$ in each hypothesis (and the required variables and parameters), we can define the variant specific and animal specific variables,

$$\vec{x}_{k,j}=\left\langle q_{k,j},f_{k} \right\rangle,$$

and the animal specific parameters

$$\vec{\theta}_{j}=\left\langle r_{j},b_{j},m_{j} \right\rangle.$$

Equation $\left( S3.3 \right)$ implies that like $r_{k,j}$, $z_{k,j}$ is a function of $\vec{x}_{k,j}$ and $\vec{\theta}_{j}$. Therefore, inserting the functional definition of $r_{k,j}$ into Equation $\left( S3.3 \right)$ gives

$$\begin{aligned} z_{k,j}\left( \vec{x}_{k,j};\vec{\theta}_{j} \right)=1-e^{-r_{k,j}\left( \vec{x}_{k,j};\vec{\theta}_{j} \right)}.\# \end{aligned}(S3.8)$$

- 1. Data and parameter optimization

To assess which formulation for $r_{k,j}$ best captures the mechanisms driving variant composition of early rebounding virus after the second ATI, we need to fit each model to data. In this subsection, we outline what data is used (i.e., variants considered, calculation of variables, and animals considered) and the fitting method.

- - 1. Tat-SL8 variants considered

In this analysis, we consider only single amino acid Tat-SL8 variants detected at least once in all sequencing done for this study and for which the minimum path is a single non-synonymous nucleotide mutation. This restriction was implemented both to limit the number of variants considered and to prevent bias against the mutation model (as shortly after reactivation, double mutants would be less likely to evolve than single mutants). Additionally, in the rare instances where two distinct single nucleotide mutations gave rise to the same amino acid sequence, those two single nucleotide mutations were designated as distinct variants. Finally, as STOP codon mutations would presumably lead to non-replication competent variants, variants with STOP codon mutations were not included in this analysis. In all, 49 variants were considered in this analysis.

- - 1. Defining variables needed for fitting

Before fitting the four models listed in Section S3.2 above, we need to define the variables $q_{k,j}$ and $f_{k}$.

Given that plasma virus is more escaped at the initiation of the second round of ART than anytime earlier, we assume the escape variant composition of replicating virus at this time point is the most representative of the escape variants present in the reservoir. Therefore, as a proxy for the relative size of individual variants in the reservoir of animal $j$, we define $q_{k,j}$ to be the proportion of the plasma virus of animal $j$ constituted by variant $k$ (after background subtraction) on the day of the second round of ART initiation (day 281 or 282).

To approximate the ease with which individual variants evolve, we assumed that for variant $k$, the propensity for evolution, $f_{k}$, could be approximated by the average proportion of the viral load on day 14 comprised of variant $k$ (after background subtraction) across all animals.

- - 1. Animals included in analysis

Animals are only analyzed if sequencing was available both on the day ART was re-initiated for the second round (to obtain $q_{k,j}$) and within 10 days of detected rebound. Additionally, animals are not included if sequencing post rebound came after CD8 depletion, resulting in a total of 8 animals who contribute to this analysis.

- - 1. Performing the model fitting

For each model listed in S3.2, we optimize the parameters, $\vec{\theta}_{j}$, for each animal using a maximum likelihood approach with the log-likelihood for animal $j$ defined by:

$$\ln\mathcal{L}_{j}\left( \vec{\theta}_{j} \right)=\sum_{k=1}^{n} d_{k,j}\ln\left( z_{k,j}\left( \vec{x}_{k,j};\vec{\theta}_{j} \right) \right)+\left( 1-d_{k,j} \right)\ln\left( 1-z_{k,j}\left( \vec{x}_{k,j};\vec{\theta}_{j} \right) \right)$$

where $n$ is the number of variants (in this work $n=49$), $\vec{x}_{k,j}$ is the set of variant and animal specific variables as defined above, and $d_{k,j}$ is an indicator variable for if variant $k$ was detected ($1$) or not ($0$) in the early rebound viral load of animal $j$.

- 1. Results

As an example of the model outputs, the best fit of the Reservoir Model is shown in Fig S3.1 for each animal (red lines) overlaid on the data for each animal. For assessment of which of the four hypotheses listed in Section S3.1 best match the early rebound data, we used both estimates of the AIC and likelihood ratio tests.


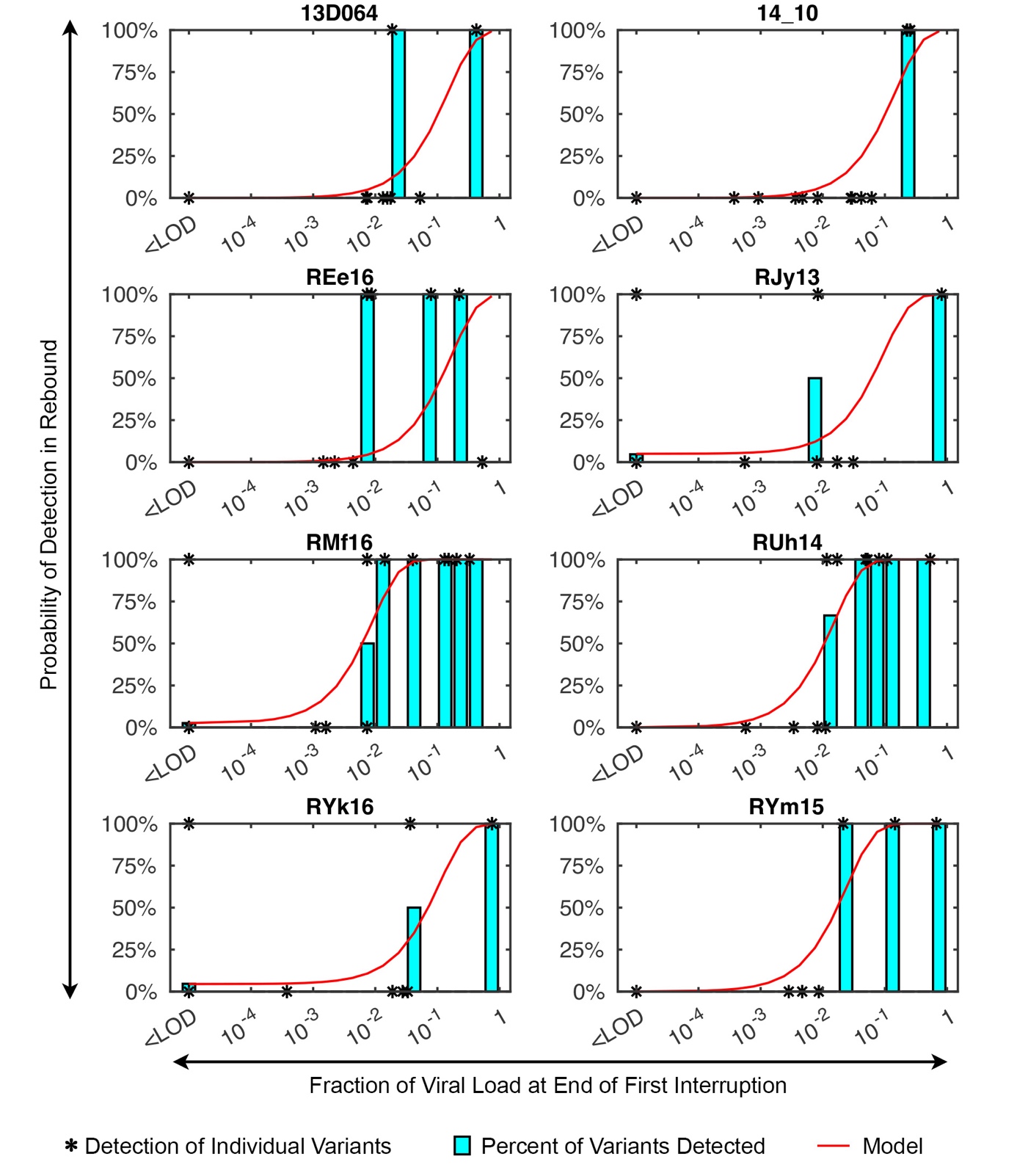


**Fig S3.1: Reservoir Model Fit.** Plotted is the best fit Reservoir Model for each of the 8 animals with sequencing at the required times. Asterisks indicate the proportion of the viral load consisting of individual variants at the start of second round of ART (horizontal axis) and whether variants were detected (100%) or not (0%) in the early rebound viral load (vertical axis). Cyan bars indicate the percentage of variants (binned along horizonal axis in .25 log_10_ increments) detected in the early rebound viral load. Red lines indicate the probability of being detected in the early rebound as predicted by the Reservoir Model with the best fit parameters for each animal.

- - 1. **Best fitting model using the AIC**

For a set of candidate models, the model with the lowest AIC ($AIC_{min}$) is the one with the most statistical support. In this analysis, we use the corrected $AIC$, $AIC_{c}$, which is given by

$$AIC_{c,j}=-2\ln\mathcal{L}_{max,j}+2h+\frac{2h^{2}+2h}{n-h-1}$$

where $\mathcal{L}_{max,j}$ is the maximum likelihood for animal $j$, $h$ is the number of model parameters, and $n=49$ is the number of data points used in the fitting (Tat-SL8 variants) (1). Note that from now on we will represent the corrected AIC ($AIC_{c}$) as $AIC$, for simplicity. For each animal $j$ and model, $M$, the difference between the corrected AIC for that model, and the minimum corrected AIC overall, $\Delta AIC_{j}^{M}=AIC_{j}^{M}-AIC_{j,min}$, was calculated, and values are listed in Table S3.1.

In 7 out of the 8 animals, the Reservoir Model has the lowest AIC. Additionally, in the remaining animal, the Combined Model has the lowest AIC, but the Reservoir Model has $\Delta AIC<2$, indicating that it has a similar level of statistical support (1). This indicates that the reservoir model is best supported by the data across the 8 animals. As further support for this conclusion, the difference between the corrected AIC of the Reservoir Model and both the Constant and Evolution Models is $>4$ in all but one animal, which generally implies the Constant and Variant Evolution Models have substantially less support (1).

Table S3.1 Model comparison by $AIC$.

| Animal | Constant Model $\Delta AIC$ | Reservoir Model $\Delta AIC$ | Evolution Model $\Delta AIC$ | Combined Model $\Delta AIC$ |
| --- | --- | --- | --- | --- |
| 13D064 | 7.91 | 0 | 7.74 | 1.65 |
| 14_10 | 16.6 | 0 | 18.8 | 2.27 |
| REe16 | 4.30 | 0 | 6.47 | 2.27 |
| RJy13 | 3.66 | 0 | 5.83 | 2.27 |
| RMf16 | 28.2 | 0 | 30.3 | 2.27 |
| RUh14 | 32.8 | 0 | 34.8 | 2.27 |
| RYk16 | 5.64 | 0.43 | 6.44 | 0 |
| RYm15 | 18.0 | 0 | 20.2 | 2.27 |

The $\Delta AIC$ values are given for each of the four models from Section S3.2 (columns) based on the data in each animal (rows).

- - 1. Best fitting model using Likelihood ratio test

In addition to the AIC analysis above, we used a likelihood ratio test to assess if there is statistical support for the reservoir and/or the evolution models. Results for each animal are listed in Table S4 below. For both the Reservoir and Evolution Models, statistical significance was assessed both independently (i.e., comparing the Reservoir or Evolution Model with the Constant Model; columns 2 and 3 of Table S3.2) and in the context where an alternative hypothesis is included (i.e., comparing the Reservoir and Evolution Model with the Combined Model; columns 4 and 5 of Table S3.2).

Likelihood ratio test results give strong support for the Reservoir Model (i.e., that Tat-SL8 variants detected early in rebound reactivated from the reservoir) and also strongly rejects the Evolution Model (i.e., that evolution after reactivation of wild-type contributes to the variants detected). Test results for inclusion of the reactivation term are always significant $(p<0.05)$, regardless of whether or not evolution is incorporated (2^nd^ and 4^th^ columns of Table S4). In fact, corresponding p-values are $<{10}^{-3}$ in 8 of the 16 comparisons. On the other hand, we see no support for inclusion of the evolution component of the model, regardless of whether or not the reservoir term is included ($p>0.05$ for all 16 comparisons in 3^rd^ and 5^th^ column of Table S4).

**Table S3.2** Model comparison by likelihood ratio test.

| Animal | Restricted Model = Constant Model | | Restricted Model = Evolution Model | Restricted Model = Reservoir Model |
| --- | --- | --- | --- | --- |
|  | Unrestricted Model = Reservoir Model | Unrestricted Model = Evolution Model | Unrestricted Model = Combined Model | |
| 13D064 | 1.49x10^-3^** | 1.26x10^-1^ | 3.82x10^-3^** | 4.31x10^-1^ |
| 14_10 | 1.46x10^-5^*** | 1 | 1.46x10^-5^*** | 1 |
| REe16 | 1.10x10^-2^* | 1 | 1.10x10^-2^* | 1 |
| RJy13 | 1.57x10^-2^* | 1 | 1.57x10^-2^* | 1 |
| RMf16 | 3.65x10^-8^*** | 8.04x10^-1^ | 3.77x10^-8^*** | 1 |
| RUh14 | 3.43x10^-9^*** | 7.00x10^-1^ | 3.70x10^-9^*** | 1 |
| RYk16 | 6.57x10^-3^** | 2.41x10^-1^ | 3.16x10^-3^** | 1.00x10^-1^ |
| RYm15 | 7.10x10^-6^*** | 1 | 7.10x10^-6^*** | 1 |

For each restricted model (first row) and unrestricted model (second row) combination, p-values for testing if the unrestricted model is a better fit are listed for each animal.

*p < 0.05, **p < 0.01, ***p < 0.001

References

1. Burnham KP, Anderson DR. Information and Likelihood Theory: A Basis for Model Selection and Inference. Model selection and multimodel inference: a practical information-theoretic approach. 2 ed. New York: Springer; 2002. p. 49-97.
