# Supplementary material for: Preferential selection of viral escape mutants by CD8+ T cell ‘sieving’ of SIV reactivation from latency
Source: PLoS Pathog. 2023 Nov 30;19(11):e1011755. doi: 10.1371/journal.ppat.1011755 (PMC10688670; doi:10.1371/journal.ppat.1011755)
Supplement: S1 Table — (DOCX) [file ppat.1011755.s004.docx]

S1 Table. Tat-SL8-specific CD8^+^ T Cell Phenotype Comparison: Prior to 1^st^ ATI vs. Prior to 2^nd^ ATI (day ~190 vs. 373-374)

| Cell Subset | Phenotype | n | Median Late ART 1  (Day ~190) | Median Late ART 2  (Day 373-4) | p-value |
| --- | --- | --- | --- | --- | --- |
| Total CD8s | Percent Tat-SL8 specific | 14 | 0.595% | 0.725% | 0.016* |
| Memory CD8s | Percent Tat-SL8 specific | 14 | 0.77% | 0.855% | 0.068 |
| Tat-SL8-Specific Memory CD8s | Percent CD69 positive | 13 | 2.33% | 2.58% | 0.22 |
|  | Percent CXCR3 positive | 13 | 66.5% | 80.5% | 0.33 |
|  | Percent CXCR5 positive | 13 | 7.36% | 15.0% | 2.4x10^-4^*** |
|  | Percent Ki67 positive | 13 | 4.66% | 2.53% | 0.0081** |
|  | Percent PD1 positive | 13 | 76.6% | 78.2% | 0.95 |
|  | Percent Perforin-Granzyme positive | 13 | 10.9% | 6.37% | 0.027* |
|  | Percent Central Memory | 13 | 69.2% | 86.3% | 7.3x10^-4^*** |
|  | Percent Effector Memory | 13 | 30.4% | 13.7% | 7.3x10^-4^*** |

Cell phenotype of Tat-SL8-specific CD8^+^ T Cells in peripheral blood was analyzed as described in S2 Text prior to the first treatment interruption (day ~190) and prior to the second treatment interruption (day 373 or 374). Based on a paired Wilcoxon signed rank test, the percent of total CD8s that were Tat-SL8 specific was significantly higher prior to the second treatment interruption. Tat-SL8 specific CD8s shifted towards the central memory phenotype and increased in CXCR5 expression prior to the second interruption, while the activation markers Ki67 and Perforin-Granzyme decreased. Shifts in other phenotypes measured were not significant.

P-values given by paired Wilcoxon signed rank test; *p < 0.05, **p < 0.01, ***p < 0.001.
