# Supplementary material for: Preferential selection of viral escape mutants by CD8+ T cell ‘sieving’ of SIV reactivation from latency
Source: PLoS Pathog. 2023 Nov 30;19(11):e1011755. doi: 10.1371/journal.ppat.1011755 (PMC10688670; doi:10.1371/journal.ppat.1011755)
Supplement: S2 Table — (DOCX) [file ppat.1011755.s005.docx]

S2 Table. Non-wild-type Tat-SL8 variants detected at greater than 20% of viral load at least once

| Wild Type | STPESANL |
| --- | --- |
| Variant 1 | P------- |
| Variant 2 | ----L--- |
| Variant 3 | -I------ |
| Variant 4 | -------R |
| Variant 5 | -------P |

The only 5 non-wild-type variants ever detected at greater than 20% of the viral load in any animal are listed above. All five of the variants listed have previously been detected in Mamu-A*01 rhesus macaques (1-4). Additionally, Allen et al. demonstrated that all five variants decreased epitope binding to MHC class 1 molecules of Mamu-A*01^+^ rhesus macaques and decreased recognition by CD8^+^ T cell lines generated from Mamu-A*01^+^ rhesus macaque PBMCs (1).

References

1. Allen TM, O'Connor DH, Jing P, Dzuris JL, Mothe BR, Vogel TU, et al. Tat-specific cytotoxic T lymphocytes select for SIV escape variants during resolution of primary viraemia. Nature. 2000;407(6802):386-90.

2. Price DA, West SM, Betts MR, Ruff LE, Brenchley JM, Ambrozak DR, et al. T cell receptor recognition motifs govern immune escape patterns in acute SIV infection. Immunity. 2004;21(6):793-803.

3. Immonen TT, Camus C, Reid C, Fennessey CM, Del Prete GQ, Davenport MP, et al. Genetically barcoded SIV reveals the emergence of escape mutations in multiple viral lineages during immune escape. Proc Natl Acad Sci U S A. 2020;117(1):494-502.

4. Moriarty RV, Golfinos AE, Gellerup DD, Schweigert H, Mathiaparanam J, Balgeman AJ, et al. The mucosal barrier and anti-viral immune responses can eliminate portions of the viral population during transmission and early viral growth. PLoS One. 2021;16(12):e0260010.
