# Supplementary material for: Preferential selection of viral escape mutants by CD8+ T cell ‘sieving’ of SIV reactivation from latency
Source: PLoS Pathog. 2023 Nov 30;19(11):e1011755. doi: 10.1371/journal.ppat.1011755 (PMC10688670; doi:10.1371/journal.ppat.1011755)
Supplement: S1 Fig — (DOCX) [file ppat.1011755.s006.docx]

**
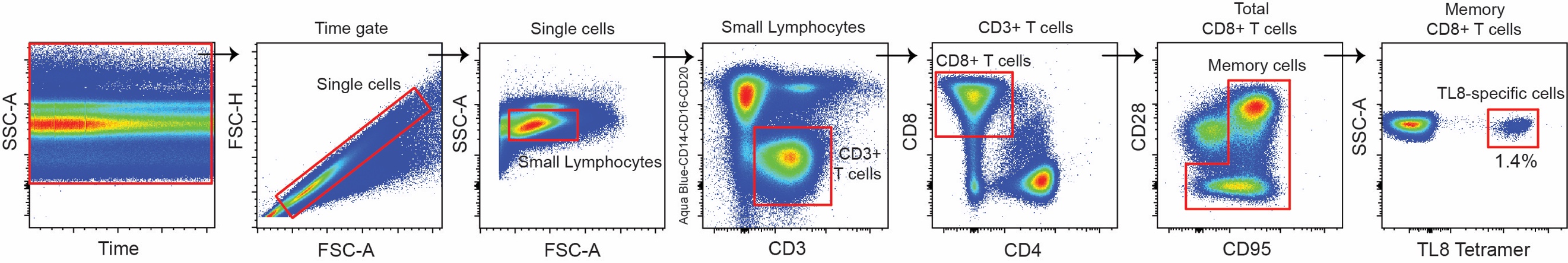
**

**S1 Fig. Gating strategy used for flow cytometric analyses.** Representative example of a rhesus macaque is shown. First, to ensure that only live single cells were analyzed from PBMCs, forward scatter height (FSC-H)-versus-forward scatter area (FSC-A) and side scatter area (SSC-A)-versus-FSC-A plots were used to exclude doublets and focus on singlet small lymphocytes. Dead cells were excluded by gating on cells negative for the viability marker Aqua Blue. Monocytes, B and NK cells were excluded via the CD14/16/20 dump gate. CD8^+^ T lymphocytes were gated within CD3^+^ T cells. SL8-specific CD8^+^ T cells were gated within total memory CD8^+^ T cells. The frequency of SL8-specific cells was calculated within total CD8^+^ T cells.
