# Supplementary material for: Preferential selection of viral escape mutants by CD8+ T cell ‘sieving’ of SIV reactivation from latency
Source: PLoS Pathog. 2023 Nov 30;19(11):e1011755. doi: 10.1371/journal.ppat.1011755 (PMC10688670; doi:10.1371/journal.ppat.1011755)
Supplement: S2 Fig — (DOCX) [file ppat.1011755.s007.docx]

**
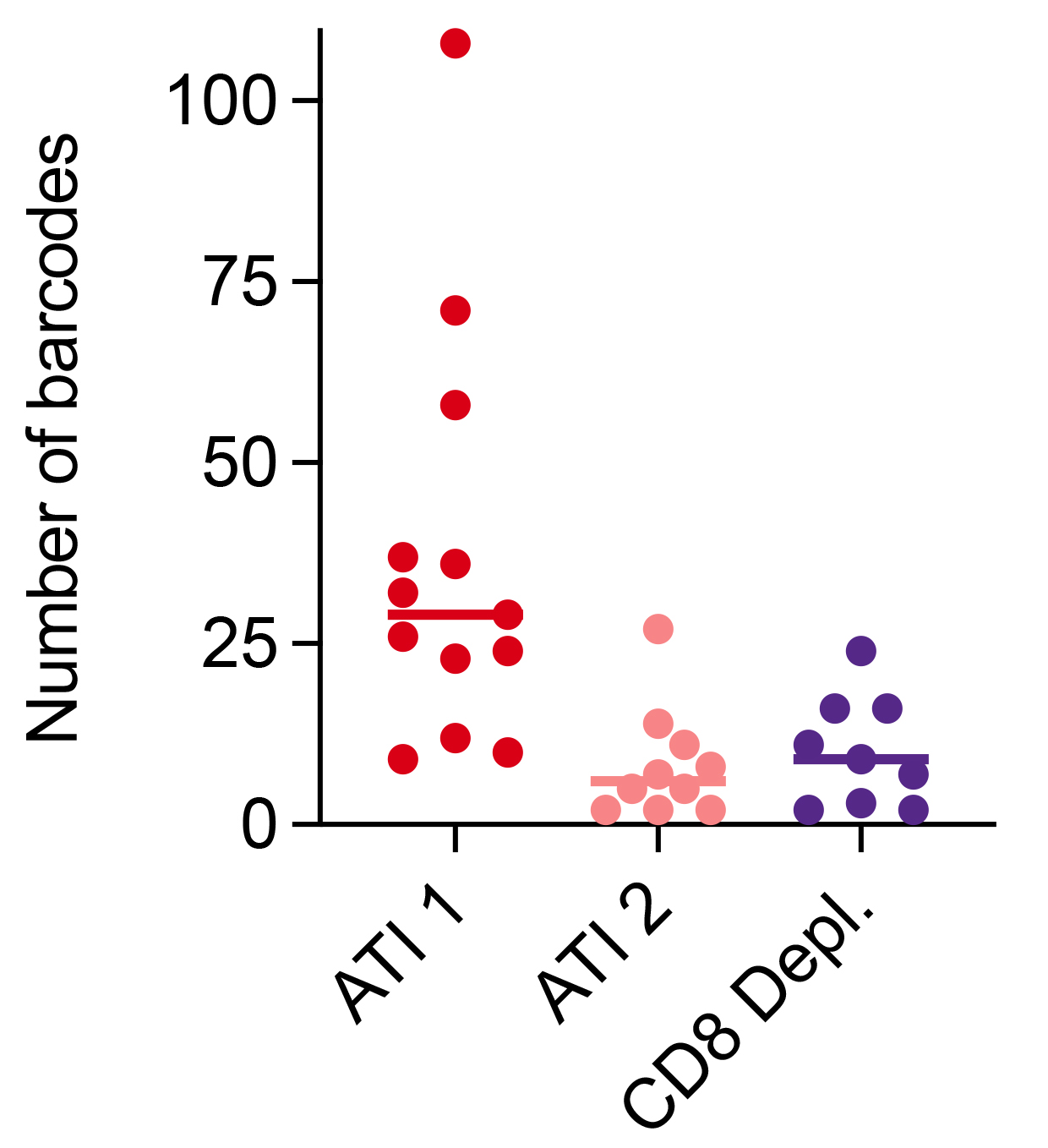
**

**S2 Fig. Rebounding barcodes.** Plotted above is the number of barcodes detected to have rebounded within individual animals after the first ATI, second ATI, and CD8 depletion. Similar to as reported for reactivation rate in the main text, number of rebounding barcodes was significantly lower post ATI 2 compared to post ATI 1 (median 6 vs. 29; p = 0.0003; paired Wilcoxon signed rank test). To compare all three time points, we use the Kruskal-Wallis test with Dunn’s multiple comparisons (as done in main text). Again, similar to reactivation rate, number of rebounding barcodes increased slightly, in a non-significant manner, following CD8 depletion (median 9 vs. 6; p > 0.99). In comparison to ATI 1, number of detected rebounding barcodes was significantly lower following CD8 depletion (p = 0.0107).
