# Supplementary material for: Preferential selection of viral escape mutants by CD8+ T cell ‘sieving’ of SIV reactivation from latency
Source: PLoS Pathog. 2023 Nov 30;19(11):e1011755. doi: 10.1371/journal.ppat.1011755 (PMC10688670; doi:10.1371/journal.ppat.1011755)
Supplement: Attachment — Submitted filename: Docken_SL8_Response_to_Reviewers.docx [file ppat.1011755.s008.docx]

We thank the reviewers for their kind comments and feedback. The reviewers’ comments are replicated below in blue text followed by our responses in black text. Note, all line numbers refer to the clean version of the manuscript.

**Reviewers' Responses to Questions**

**Part II – Major Issues: Key Experiments Required for Acceptance**

No ‘Major Issues’ were identified by the reviewers.

**Part III – Minor Issues: Editorial and Data Presentation Modifications**

**Reviewer #1:** I found that the calculation of "viral reactivation rate" was confusing and unnecessary for the message of the paper. When I tried to understand this calculation I found the authors mainly referred to a previous paper, which is very annoying for a reader. Importantly, they did not even summarize the assumptions that went into the calculation (which definitely should be included in a revision). I tried to briefly look at the referenced papers and it seems the calculation requires assuming that reactivation events are evenly spaced, and that the growth rates of all strains identical. Both these seem unlikely to be true, but the second especially ... the whole point of this paper is showing that growth rate of some non-escape strains is so much lower than that of escape strains that none are detected at all! So naturally this would not be a binary thing but likely continuous and there could be a while distribution of rates. I thought the paper would be stronger if the authors used some measure of number of reactivating virus or other quantity extracted from the barcodes that was more data-driven and less reliant on an unclear and perhaps overly restrictive model.

We thank the reviewer for their comments and apologise if the methods were unclear. To make the methodology of our reactivation rate estimates clearer, we have lengthened the description and listed the primary assumptions used at the first point where reactivation rate estimates are mentioned (lines 175-181) as well as added detail to the Materials and Methods (lines 675-678).

It is perhaps worth clarifying that the method does not assume that reactivation events are evenly spaced (it just estimates the average spacing) and although it assumes similar growth rates of founders, it is generally robust to distributions in growth rates (based on unpublished simulation analysis). However, we agree that the paper would be stronger with the addition of a direct measure of reactivating barcodes. We have now added a new supplemental figure (S2 Fig.) illustrating the number of detected barcodes after ATI 1, ATI 2, and CD8 depletion, as suggested. Similar qualitative results are observed in the total number of detected barcodes as the reactivation rate estimates, and this analysis (including S2 Fig.) is referenced when reactivation rate comparisons are mentioned in the main text (193-195, and 418-421). We agree that including the number of successful reactivating barcodes in addition to reactivation rate estimates strengthens the manuscript.

In a similar vein, I also thought it would be more clear if the authors reminded readers somewhere that their measures of # of reactivating lineages were only those surviving, and that many others could have died out by stochastic extinction before detection.

The reviewer correctly points out that our sequencing would not identify clonotypes that did not grow to the level of detection in vivo. To clearly highlight this, we have added the following sentences to the Results and Discussion.

In the Results (lines 196 to 199),

*“We note that our sequencing only identified barcoded clonotypes that successfully expanded to the level of detection by sequencing. Therefore, it is possible many more barcodes initially reactivated from latency following ATI but subsequently failed to grow to the level of detection.”*

In the Discussion (lines 441 to 446),

*“Given sequencing only identified clonotypes that had successfully grown to the level of detection by sequencing, other latently infected cells may have reactivated during the ATIs but either died out or failed to grow to the level of detection. It is not possible to differentiate whether CD8^+^ T cell “sieving” of clonotypes occurs via early killing of the first reactivating cell, or control of subsequent viral replication such that a clonotype did not reach the level of detection.”*

In addition I thought it was an interesting observation that even though their was more evidence of CTL escape at the second ATI, there was still lower peak and set point viral load than the first ATI, suggesting that overall control of viremia was higher, perhaps due to the combined effect of a fitness cost of the escape mutation(s) and the residual CTL control. This is important because people might (falsely) assume that using future immunotherapies to control rebound could make things worse than no treatment if they select for escape (like people sometimes think about drug resistance). Of course this might be case specific but it clearly doesn't seem like the case here.

We thank the reviewer for highlighting and we agree this is important to clarify. We have added the following text to lines 468 to 474 of the Discussion to draw the reader’s attention to this point,

*“Interestingly, the high frequency of immune escape from CD8 pressure during the second ATI did not lead to reduced virologic control compared to the first ATI. In fact, both peak and set point viral load were significantly lower at the second ATI than the first, potentially due to alternative means of immune pressure or the fitness costs of escape mutation [1]. Importantly, this suggests that immune escape during an ATI does not necessarily imply reduced immunologic control of viral replication during subsequent treatment interruptions.”*

I thought the authors could give a little bit more background on this particular macaque model they were using and why they feel they can assume escape vs non-escape strain based on a single epitope, as readers unfamiliar with that literature would be a little lost here.

We have given a more in-depth description of the CD8 pressure on Tat-SL8 in Mamu*A01^+^ rhesus macaques and the evidence that this pressure is specific to just the Tat-SL8 epitope (lines 107 - 115).

*“The barcode site is just upstream of the Tat-SL8 epitope, which has been well characterized as a locus of CD8 pressure early in SIV infection of Mamu*A01^+^ RMs [2-4]. Tat-SL8-specific CD8^+^ T cells have routinely been identified through single peptide stimulation, indicating the strong specificity of these cells [2-4], and it has been shown that addition of Tat-SL8 specific CD8^+^ T cells substantially inhibits SIVmac239 replication in vitro [4]. Due to the proximity of Tat-SL8 to the barcoded region of SIVmac239M, both can be sequenced simultaneously via high throughput Illumina sequencing, allowing studies of immune escape on individual viral barcode lineages.”*

This is probably a better fit for future work, but I also thought the study would be a good opportunity to do some modeling to provide some quantitative estimate of the cost of escape/strength of CTL response, rate of CTL expansion, etc.

We have previously performed a number of studies characterising the rates of immune escape and reversion of SIV in vivo (and agree this is an interesting topic) [1, 5-7]. However, given that much work has already been done on the topic of strength of selection (and the question is somewhat peripheral to our focus here), we feel it may confuse rather than clarify to add this analysis here.

Finally, in this day and age I don't think it's acceptable to submit a paper for review with a data/code availability statement saying it will be shared upon publication. This should be part of the review process. When I was looking back at the authors' previous paper in this same journal to see the methods about the rebound virus (Fennessy 2017) I noticed there they mistakenly claim their data is available in the SI but it is not, so these resources need to be confirmed by reviewers/editors pre-publication.

We apologise that this was not available for review. A link to the GitHub repository, where data and code for this work are fully available, is provided below. To clone this repository, click ‘Clone’ on the website and copy the URL that appears. Then use GitHub/GitHub Desktop’s ‘Clone a Repository’ functionality via the URL. Upon publication, this repository will be made public with a link to the repository included in the manuscript.

https://gitfront.io/r/user-6637481/9ypxo4u6yc2P/CD8-sieving-TatSL8-PlosPath2023/

**Reviewer #2:** The abstract is unnecessarily short for this journal, and leaves out important details of the study. This is a minor criticism at best.

We thank the reviewer for this suggestion. We have lengthened the abstract and included more detailed descriptions of the study and our findings.

**Reviewer #3:** Figure 1: Sub-figures C, E, and G are of a poor quality. Please enhance the quality of the light and dark green dots and lines.

To improve the quality of Figure 1C, E, and G (now C, D, and F), we have increased the size of the dots, outlined the filled dots in black, and changed the blue used for cell associated DNA to cyan.

Figure 1B caption 1: There is a typo in line 125.

We thank the reviewer for highlighting this typo. It has been corrected.

Line 146: Since FTY720 had no effect on the rebound characteristics, was there any difference between FTY720 and control group? Has it been previously shown that the FTY720 had a limited or no effect? Maybe you could add a reference.

FTY720 had the expected effect of greatly reducing circulating CD4^+^ and CD8^+^ T cell numbers. Previous work in early SIV infection had shown FTY720 during early ART to have minimal effects on either early viral kinetics or subsequent rebound kinetics [8]. We believe ours is the first study to directly analyse the effects of FTY720 during rebound. However, this is somewhat peripheral to the questions addressed in the present manuscript. A manuscript is currently in preparation specifically addressing the effects of FTY720 on T cell numbers and the lack of effect on viral kinetics. This work has been presented at the 37^th^ Annual Symposium on Nonhuman Primate Models for AIDS (P13 in [9]).

We have added the reference to the previous manuscript in lines 167-171,

*“Notably, FTY720 had no significant effect on any of the viral rebound parameters used for our analyses of viral escape dynamics (similar to as reported for FTY720 treatment during early ART [8]); as a result, the FTY720-treated and control animals were combined for this study.”*

Figure 2: It seems that the mutant increases in two phases: initially fast and later slow. What do you think drives the two-phasic increase in mutant virus? Maybe you can write one or two sentences in the manuscript explaining the phenomenon.

This is an intriguing feature of the data, and it is unclear what mechanism would allow wild-type virus to persist at such low levels. We have added the following text to lines 250 to 254 to highlight this interesting feature of the data,

*“Furthermore, in both this and previous studies, decline in wild-type prevalence slowed after dropping to near 10% of the viral load and then appeared to persist in some animals at approximately 1% of the plasma virus. It is unclear why wild-type virus persists at these low levels despite the clear competitive advantage of escaped variants.”*

Line 513/514: You could refer to Figure 1B.

We have added the text *“(schematically illustrated in Fig. 1B)”* to the text as suggested.

Line 562/563: closing bracket is missing.

We added the missing closing bracket in line 721.

**References:**

1.	Loh L, Batten CJ, Petravic J, Davenport MP, Kent SJ. In vivo fitness costs of different Gag CD8 T-cell escape mutant simian-human immunodeficiency viruses for macaques. J Virol. 2007;81(10):5418-22. Epub 20070307. doi: 10.1128/JVI.02763-06. PubMed PMID: 17344299; PubMed Central PMCID: PMCPMC1900239.

2.	Allen TM, O'Connor DH, Jing P, Dzuris JL, Mothe BR, Vogel TU, et al. Tat-specific cytotoxic T lymphocytes select for SIV escape variants during resolution of primary viraemia. Nature. 2000;407(6802):386-90. Epub 2000/10/03. doi: 10.1038/35030124. PubMed PMID: 11014195.

3.	Price DA, West SM, Betts MR, Ruff LE, Brenchley JM, Ambrozak DR, et al. T cell receptor recognition motifs govern immune escape patterns in acute SIV infection. Immunity. 2004;21(6):793-803. doi: 10.1016/j.immuni.2004.10.010. PubMed PMID: 15589168.

4.	Loffredo JT, Burwitz BJ, Rakasz EG, Spencer SP, Stephany JJ, Vela JP, et al. The antiviral efficacy of simian immunodeficiency virus-specific CD8+ T cells is unrelated to epitope specificity and is abrogated by viral escape. J Virol. 2007;81(6):2624-34. Epub 2006/12/29. doi: 10.1128/JVI.01912-06. PubMed PMID: 17192314; PubMed Central PMCID: PMCPMC1866004.

5.	Davenport MP, Loh L, Petravic J, Kent SJ. Rates of HIV immune escape and reversion: implications for vaccination. Trends Microbiol. 2008;16(12):561-6. Epub 20081027. doi: 10.1016/j.tim.2008.09.001. PubMed PMID: 18964018.

6.	Fernandez CS, Stratov I, De Rose R, Walsh K, Dale CJ, Smith MZ, et al. Rapid viral escape at an immunodominant simian-human immunodeficiency virus cytotoxic T-lymphocyte epitope exacts a dramatic fitness cost. J Virol. 2005;79(9):5721-31. doi: 10.1128/JVI.79.9.5721-5731.2005. PubMed PMID: 15827187; PubMed Central PMCID: PMCPMC1082732.

7.	Kent SJ, Fernandez CS, Dale CJ, Davenport MP. Reversion of immune escape HIV variants upon transmission: insights into effective viral immunity. Trends Microbiol. 2005;13(6):243-6. doi: 10.1016/j.tim.2005.03.011. PubMed PMID: 15936652.

8.	Pino M, Pagliuzza A, Pampena MB, Deleage C, Viox EG, Nguyen K, et al. Limited impact of fingolimod treatment during the initial weeks of ART in SIV-infected rhesus macaques. Nat Commun. 2022;13(1):5055. Epub 2022/08/28. doi: 10.1038/s41467-022-32698-y. PubMed PMID: 36030289; PubMed Central PMCID: PMCPMC9420154.

9.	Oral Presentations. Journal of Medical Primatology. 2022;51(5):292-326. doi: 10.1111/jmp.12612.
